# Establishing a Pharmacoinformatics Repository of Approved Medicines: A Database to Support Drug Product Development

**DOI:** 10.1021/acs.molpharmaceut.4c00991

**Published:** 2024-12-20

**Authors:** Jack D. Murray, Harriet Bennett-Lenane, Patrick J. O’Dwyer, Brendan T. Griffin

**Affiliations:** School of Pharmacy, University College Cork, College Road, Cork T12 K8AF, Ireland

**Keywords:** cheminformatics, machine learning, computational pharmaceutics, drug product database, absorption, formulation

## Abstract

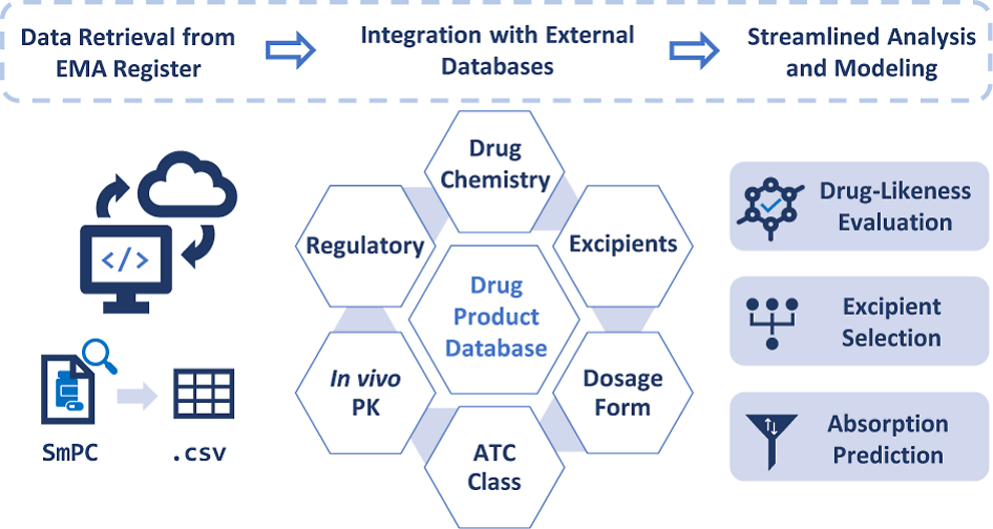

Advanced predictive modeling approaches have harnessed data to fuel important innovations at all stages of drug development. However, the need for a machine-readable drug product library which consolidates many aspects of formulation design and performance remains largely unmet. This study presents a scripted, reproducible approach to database curation and explores its potential to streamline oral medicine development. The Product Information files for all centrally authorized drug products containing a small molecule active ingredient were retrieved programmatically from the European Medicines Agency Web site. Text processing isolated relevant information, including the maximum clinical dose, dosage form, route of administration, excipients, and pharmacokinetic performance. Chemical and bioactivity data were integrated through automated linking to external curated databases. The capability of this database to inform oral medicine development was assessed in the context of drug-likeness evaluation, excipient selection, and prediction of oral fraction absorbed. Existing filters of drug-likeness, such as the Rule of Five, were found to poorly capture the chemical space of marketed oral drug products. Association rule learning identified frequent patterns in tablet formulation compositions that can be used to establish excipient combinations that have seen clinical success. Binary prediction models of oral fraction absorbed constructed exclusively from regulatory data achieved acceptable performance (balanced accuracy_test_ = 0.725), demonstrating its modelability and potential for use during early stage molecule prioritization tasks. This study illustrates the impact of highly linked drug product data in accelerating clinical translation and underlines the ongoing need for accuracy and completeness of data reported in the regulatory datasphere.

## Introduction

Machine learning (ML) is poised to drive the efficiency of the pharmaceutical industry by facilitating data-powered decisions throughout the medicine development lifecycle.^1,2^ Data is the currency of ML, yet existing sources of data do not adequately support drug product development. The total amount of data, known as the datasphere, grows exponentially year-on-year, and has far exceeded the capacity for human analysis. In the context of pharmaceutical research, this data may encompass regulatory documents, peer-reviewed articles, and large databases of drug-like molecules. This data is often unstructured, heterogeneous, and requires significant curation before it can be used for predictive modeling to optimize product development.^3,4^ In parallel, the drive toward the Pharma 4.0 operating model emphasizes the need for digital maturity and data integrity by design to accelerate the digital transformation of pharmaceutical manufacturing.^5,6^ Pharmacoinformatics describes the application of computational and informatics approaches to all aspects of the drug development pipeline, from discovery to postmarketing surveillance.^7,8^ As the pursuit of novel small molecule drugs expands further beyond the established Rule of Five (Ro5) chemical space,^9,10^ challenges relating to lead selection and formulation may be better met by a computational approach rather than traditional experimentation.^11^ In this regard, efficient methods of extracting machine-readable data from the datasphere are needed to support future modeling efforts.^12^

Formulation design lacks the large, labeled data available for other tasks in the product development lifecycle, such as bioactivity prediction.^8^ Consequently, the prevailing approach to ML-informed formulation design has been to leverage limited experimental data sets specifically obtained for predictive modeling.^4^ This strategy may be necessary for certain applications, but data from existing products is likely to be broader and have formulation-specific in vivo data available, notwithstanding a bias against new chemical entities. The overwhelming majority of public databases, including PubChem,^13^ DrugBank,^14^ and ChEMBL,^15^ are drug substance-centered rather than drug product-centered, and do not link drug molecule chemistry, excipients, dosage form, route of administration, and pharmacokinetic performance in a single drug product library. PubChem^13^ provides detailed structural and chemical information for both active substances and excipients with a discrete chemistry, but does not consider pharmacokinetic information. DrugBank^14^ offers pharmacokinetic and physicochemical data, yet product-specific information, such as excipients included in formulations, is lacking. A more recent database curated by Li et al.^16^ comprehensively links drug substance properties with pharmacokinetic properties in a standardized format but again considers the drug molecule rather than the drug product.

Approaches to database construction for drug product development and pharmacokinetic prediction have been largely manual, reflecting the unstructured nature of the pharmacoinformatics datasphere. Lombardo et al.^17^ manually searched the literature to obtain pharmacokinetic parameters for 1352 intravenously administered small molecules, which has since been successfully used for predictive modeling.^18−20^ Dubbelboer and Sjögren^21^ described the manual curation of a database of subcutaneously administered products from the regulatory literature. The researchers highlight the potential of their database to drive in silico modeling of the pharmacokinetic profiles of subcutaneously administered drugs. Zaslavsky and Allen^22^ adopt a similar approach to data collection in developing a data set to support the development of self-emulsifying drug delivery systems. While natural language processing (NLP) may facilitate automated database construction, the need for high-fidelity data retrieval and curation usually necessitates the need for human verification. To ensure the accuracy of DrugBank,^14^ data extracted by NLP is verified by a human expert before being added to the database. The error rate of curated drug databases remains largely unexplored, and erroneous data can result in significant error propagation. Tiikkainen et al.^23^ compared entries in three large bioactivity databases and found discrepancies in small molecule structure to be the most prevalent error. Dubbelboer and Sjögren^21^ noted in the curation of their own database that the DrugBank measured log *P* for venetoclax and nabumetone at the time of writing were 99 and 2400 respectively, and therefore placed an upper cap of 10 on log *P* values for the purpose of analysis. Esaki et al.^24^ further demonstrated that the accuracy of ML models of intrinsic clearance can be improved through extensive curation of publicly available data. In addition, attempts at data integration through consortia, such as Open PHACTS^25^ and UNICOM,^26^ and pointer databases, such as UniChem,^27^ further support the interoperability of the pharmacoinformatics datasphere.

By contrast, while the regulatory data landscape is largely product-centered, it is designed to meet the needs of clinicians and marketing authorization holders. Although statistical analyses of regulatory databases with a focus on drug products have been reported,^28^ information is largely optimized for human-readability rather than machine-readability. Regulators, including the European Medicines Agency (EMA) and the United States Food and Drug Administration (FDA), are currently implementing the International Organization for Standardization (ISO) standards for the Identification of Medicinal Products (IDMP).^29^ The five ISO IDMP standards (namely substance identification, pharmaceutical dosage forms, units of measurement, medicinal product identification, and pharmaceutical product identification) are designed to ensure the interoperability and transparency of data relating to drug products.^30^ The potential benefits of a single global data model for drug product information are obvious for all stages of the drug product development lifecycle.^31^ The precise implementation of the ISO IDMP standards, however, will determine whether this data architecture is sufficient to support predictive modeling tasks, such as formulation design.

In the European context, the EMA is complying with ISO IDMP standards through the Substance, Product, Organization, and Referentials (SPOR) data management services.^32^ Taking the Substances Management Services (SMS) as an example, all substances which are included in drug products authorized by the EMA have a corresponding entry in the SMS central dictionary. In most cases, substances are then linked to their molecular formula, International Chemical Identifier (InChI) Key (InChIKey),^33^ and the FDA’s Unique Ingredient Identifier (UII). The SMS greatly mitigates the requirement for manual curation despite several invalid and duplicate entries, but the SPOR system as a whole does not link these substance properties with in vivo pharmacokinetic performance, as pharmacokinetic parameters are not required fields in the Product Management Service (PMS).^32^ Electronic formats of statutory documents, such as the Summary of Product Characteristics (SmPC), partially achieve this goal, but this data still requires human or NLP curation due to the semistructured nature in which information is reported.^34,35^ This limits the utility of the EMA electronic Product Information (ePI) infrastructure for formulation optimization and pharmacokinetic prediction.^36^ Although NLP has been exploited to analyze the semantic distance between European Public Assessment Reports (EPARs) and EMA scientific guidelines, the focus in that instance was textual similarity rather than information retrieval.^37^

The aim of this research was to construct a drug product library that links the physicochemical and formulation properties of small molecule drug products with in vivo performance using computer programming. To achieve this, an EPAR Product Information file for all drug products containing a small molecule active ingredient was downloaded from the EMA central register. A combination of automated and manual processing was used to split the semistructured files into their components and extract the required information. All drugs and excipients with a discrete chemical structure were matched to their chemical identifiers and exploratory analysis of the final database was performed using Python. Potential impacts of this database include the early identification of trends in newly authorized medicines, and the development of predictive in silico models that link medicine design with in vivo performance.

## Methods

### Computational Environment

All code was authored and executed using Jupyter Notebook (version 7.0.8) with Python 3.12.4. Custom functions for database construction were organized into the constructiontools.py file to enhance readability. The packages used are included in the requirements.txt file. The computational environment consisted of a personal laptop running Windows 11 powered by an Intel Core i7-1255U processor. The code associated with this study and an interactive web app are available via the GitHub repository: https://github.com/jack-d-murray/pharmacoinformatics.

### Data Sources

#### EMA Register

The table of all EPARs for human and veterinary medicines was downloaded from the EMA Web site on the second of January 2024. This contains information on all medicines authorized via the centralized procedure by the EMA.^38^

#### EMA SMS

The SMS central dictionary was downloaded from the EMA Web site on the second of January 2024. This dictionary maps substance name to various other fields, including domain (human or veterinary), substance type (polymer, chemical, structurally diverse, etc.), and various identifiers (e.g., InChIKey).

#### ChEMBL

ChEMBL is a manually curated resource that integrates the chemistry, biological activity, and genomic research associated with bioactive, drug-like compounds.^15^ ChEMBL allows for programmatic data retrieval, and was therefore used to integrate bioactivity data associated with both drugs and excipients captured in the pharmacoinformatics database.^39,40^

#### PubChem

PubChem is the largest open-source database of chemical information. PubChem was used to source the chemical structure for all active ingredients and excipients with a discrete structure. Python libraries and programmatic access were employed to retrieve data from the current web version of the PubChem database.^41^

#### Lombardo et al. 2018 Intravenous Pharmacokinetics Database

The Lombardo et al.^17^ database contains the drug name, SMILES string, InChIKey, and five human intravenous pharmacokinetic parameters (volume of distribution at steady state, human clearance, fraction unbound, mean residence time, and terminal half-life) for 1352 drugs. This database was used to source pharmacokinetic parameters where available.

### Database Construction

**Figure 1:**
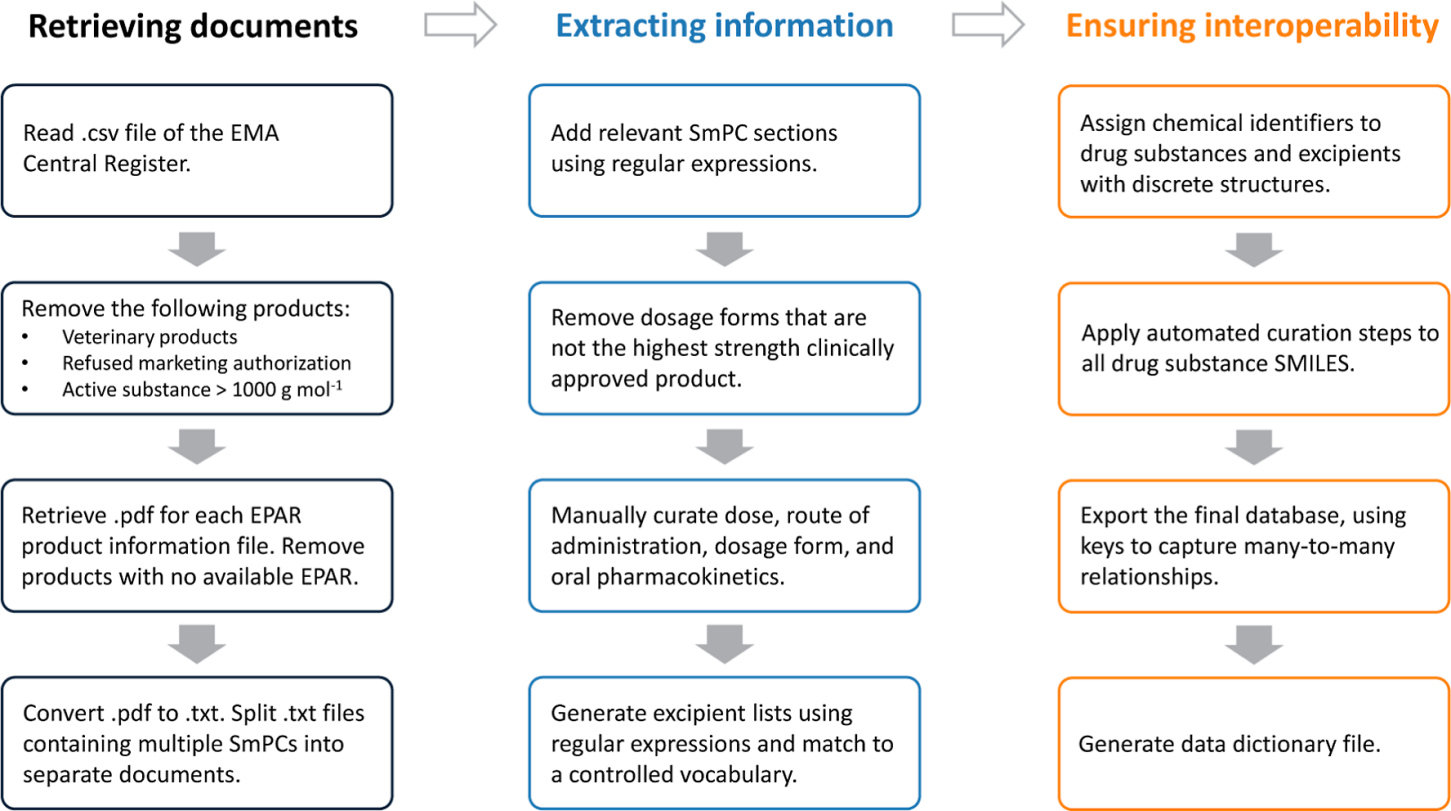
Flowchart summarizing the database construction process.

The following sections provide an overview of the database construction process, which is also summarized in Figure 1. The reader is directed to the GitHub repository for a complete description of database construction.

#### Downloading Product Information

The EMA Central Register and SMS were downloaded from the EMA Web site. Custom functions from the constructiontools.py module were used to remove products that were not of interest, including veterinary products and biologics. All active substances were matched to their corresponding InChIKey as recorded in the SMS. As the InChIKey is a hashed representation of the InChI string, it cannot be used to algorithmically regenerate the structure. The REpresentational State Transfer (REST)-style version of the PubChem Power User Gateway (PUG) was used to obtain the InChI string from the InChIKey via programmatic access.^41^ Products containing an active substance whose parent form has a molecular weight above 1000 g mol^–1^ as calculated using RDKit^42^ were excluded.

Each product in the EMA Central Register has a corresponding Uniform Resource Locator (URL) to its page on the EMA Web site. Web scraping libraries were employed to download each product’s EPAR Product Information .pdf file and assign them systematic names. The product’s page was manually inspected where no such file was returned. As each document may contain more than one SmPC, the .pdf files were converted to plain text format and programmatically split into their component SmPCs. The following SmPC sections were then added to each row of the table using regular expression searching: 2. Qualitative and Quantitative composition, 3. Pharmaceutical Form, 5.2. Pharmacokinetic Properties, and 6.1. List of Excipients.

#### Dosage Form and Route of Administration

A controlled vocabulary was used to define the allowed values for both dosage form and route of administration. For every brand, only the highest-strength clinical formulation was selected to be included in the database for each dosage form marketed. All licensed routes of administration for each formulation were listed. In the case of Paxlovid, which contains separate tablets of nirmatrelvir and ritonavir, the product was split over two rows to allow each tablet to be treated as a separate formulation. In contrast, only the netupitant component of Akynzeo hard capsules was considered, given that the palonosetron component is already accounted for in a separate product, Aloxi soft capsules.

#### Active Substances

The active substances field in the EMA Table of EPARs did not accurately reflect the correct salt and solvate form of drugs in all cases. As such, the correct form of each active ingredient was manually obtained from the Qualitative and Quantitative Composition section of the SmPC. The dose(s) of each active ingredient in the highest strength of each formulation was added to the dose column using standardized units. The form of each drug that the doses refer to were specified. Working with common names for drug molecules presents significant challenges in terms of obtaining the corresponding chemical structure in a reliable and automated manner. In instances where the active substance had an entry in the SMS central dictionary, the InChIKey provided was used to retrieve the isomeric SMILES string and InChI string using programmatic access to PubChem. Alternatively, active ingredients without a match were assigned identifiers and structures using PubChemPy, a wrapper around the PubChem PUG REST application programming interface.

Curation pipelines were applied to programmatically standardize and remove salt/solvent moieties from the structure. Converting between different line notations when generating the parent compound from a structure with nonbonded moieties can introduce unexpected errors. For example, converting the InChI string representation to an isomeric SMILES representation of pemetrexed disodium hemipentahydrate via an RDKit Mol object may result in four negative charges being assigned to one pemetrexed molecule, rather than two equivalent dianionic pemetrexed molecules. Similarly, the form of the drug that should be used is task-dependent, and an automated method of removing salts and solvents is therefore necessary. While a representation that includes all parts of a multicomponent system would be necessary to search for crystallographic data, only the parent drug is generally considered for bioactivity prediction. Additionally, certain tasks require the selective removal of solvent moieties but the retention of counterions, such as to calculate molecular weight in the same manner as Lipinski et al.^9^ in some sections of the original Ro5 analysis (where the term “formula weight” is used).

**Figure 2:**
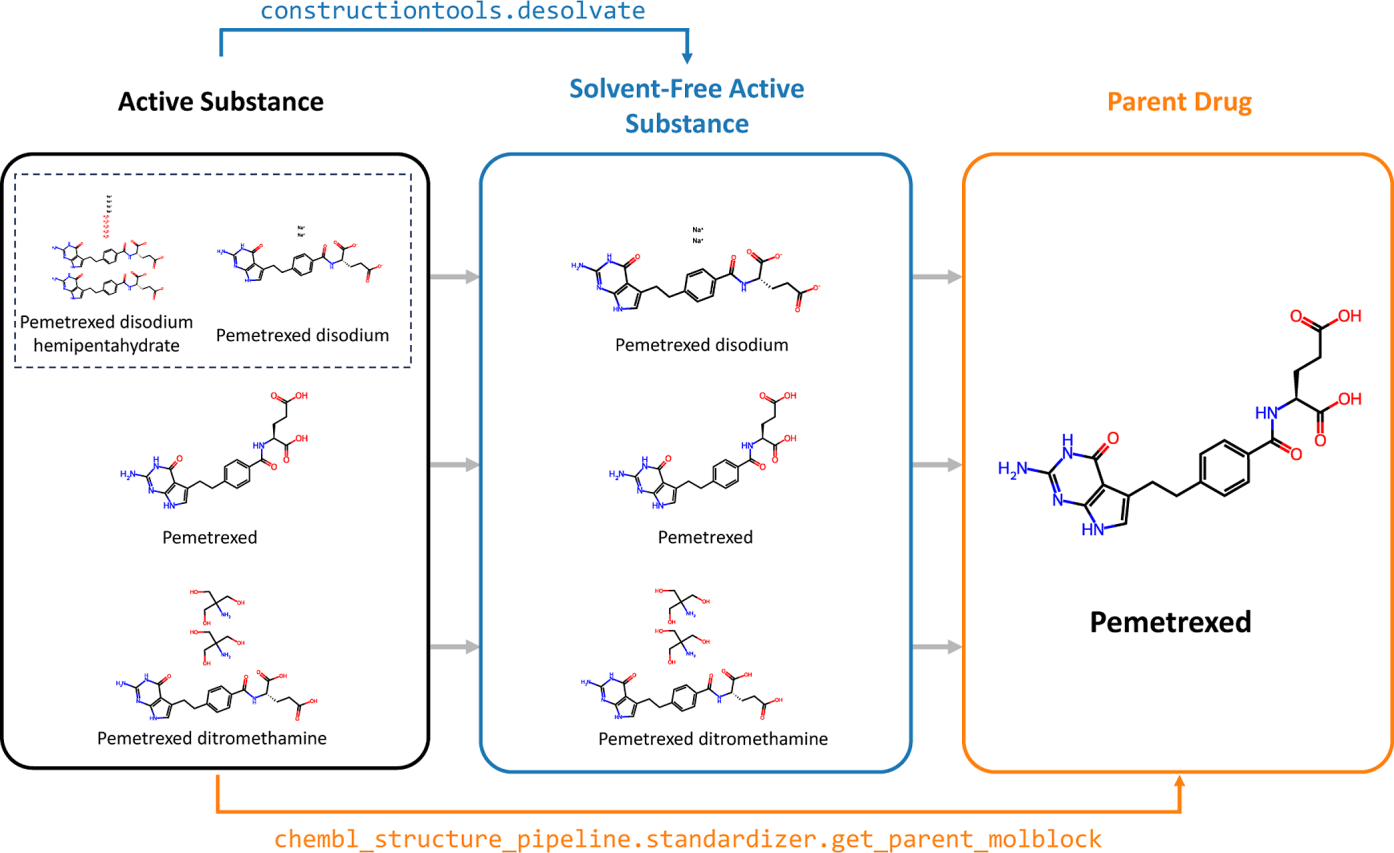
Flowchart demonstrating the automated curation steps applied to all drug substances. The get_parent_molblock method from ChEMBL’s curation protocol^43^ was used to retrieve the parent molecule, pemetrexed, successfully from four different formulated presentations of the drug. Note that the dihydrate form of pemetrexed ditromethamine also appears in the SMS central dictionary but was not mentioned in any product information file.

Figure 2 summarizes the curation pipelines employed to convert a formulated active substance with salt and solvate moieties to its parent form, and also a form where only the solvent moieties have been removed. The parent form of the active ingredient was generated by applying the GetParent module from the ChEMBL structure pipeline library to an isomeric SMILES string, which was manually corrected where necessary.^43^ A custom function which removed solvent moieties and made other appropriate adjustments to the original SMILES string was used to generate a form that contains only the parent drug and any counterions or coformers. The component with the longest SMILES in the final output was selected as the active ingredient. A full description of this function is available on the GitHub repository. While this function was unable to distinguish between salts and other possible solid forms, such as cocrystals,^44^ and has the potential to perceive an inactive moiety with a high molecular weight as the active ingredient, it was nonetheless successful in generating a valid SMILES string for almost all drug substances without manual curation. Sacubitril/valsartan, the only drug–drug cocrystal in the database, was treated as two separate free drugs to simplify analysis.

#### Formulation

Regular expressions were used to obtain a textual representation of the excipients included in each product. Various automated and manual processing steps were applied to convert each string to list of excipients restricted to a curated vocabulary of 364 allowed values. Rather than adhering strictly to chemical names for excipients, an emphasis was placed on the need for complexity reduction. As such, classes of polymers which are typically associated with a viscosity and/or molecular weight (e.g., polyethylene glycol) were grouped into single identifiers. Similar approaches were adopted with other classes of excipients where rational. Finally, all excipients with a discrete chemical structure were matched to identifiers using the same methodology described for active substances.

#### Pharmacokinetics

The pharmacokinetic parameters were divided into formulation-dependent parameters and formulation-independent parameters. Three formulation-dependent parameters, namely fraction absorbed (*F*_a_), bioavailability (% *F*), and time to reach maximum plasma concentration (*T*_max_), were obtained for oral products only from the relevant SmPC section. Parameters obtained from single-dose studies in healthy fasted volunteers were selected where available, but in most cases inference of values for *F*_a_ from human mass balance studies was necessary. Where a range of values was provided without an appropriate measure of central tendency, the arithmetic mean of the upper and lower limits was selected. Five formulation-independent pharmacokinetic parameters, namely volume of distribution at steady state (Vd_ss_), fraction unbound in plasma (*f*_u_), mean residence time (MRT), terminal half-life (*t*_1/2_), and whole-body clearance (Cl), were sourced from the Lombardo et al.^17^ Human Intravenous Pharmacokinetic Parameters database where available. Missing values were not imputed by any computational means.

#### Bioactivity

The ChEMBL ID for each drug and excipient with a discrete structure was retrieved using the ChEMBL web resource client,^39^ with the InChIKey as the filter.

#### Database Structure

**Figure 3:**
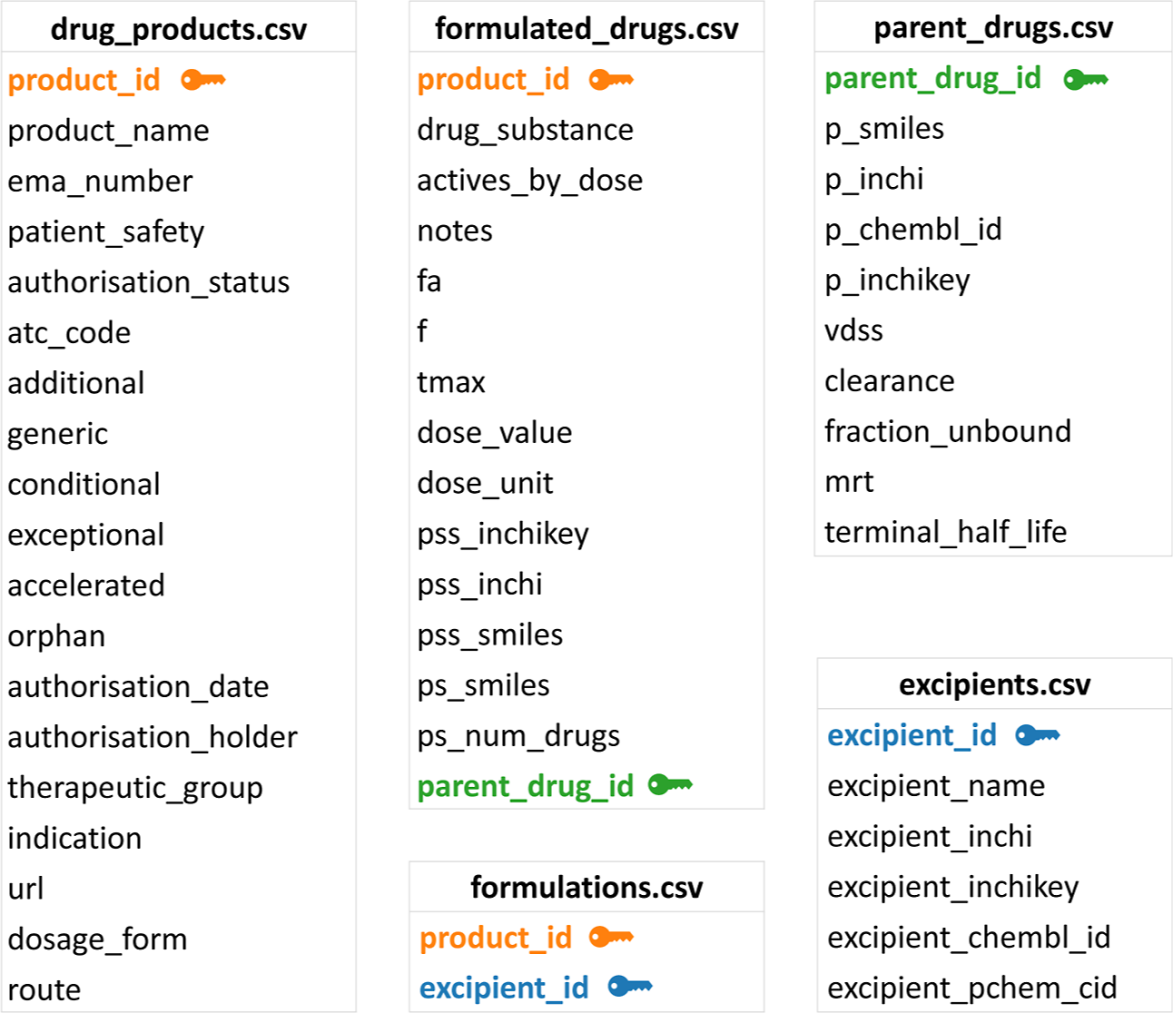
Schema of the final database. The reader is directed to the GitHub repository for a full description of each data field in the data dictionary.

The final database is reported as five .csv files on the GitHub repository. The schema of this database is shown in Figure 3. Given the large number of many-to-many relationships, a relational structure was chosen to minimize redundancy through the use of junction tables, such as formulations.csv. This also allows for the separation of formulation-dependent and formulation-independent pharmacokinetic parameters. While not all redundancy is removed by this structure, the advantages in terms of interpretability over a strictly relational structure are obvious.

### Assessment of Drug-Likeness

The chemical space of orally administered drugs in our database was compared against that of all oral drugs which have reached Phase IV of development on ChEMBL. *t*-distributed Stochastic Neighbor Embedding (*t*-SNE)^45^ was applied to the pairwise Tanimoto distances^46^ of all drugs as measured using Molecular ACCess System (MACCS) keys^47^ to generate a structure-based projection of chemical space. The use of *t*-SNE to explore the chemical diversity of one database in relation to another has been described previously.^48,49^ Principal component analysis (PCA) was applied to all normalized RDKit Lipinski descriptors to visualize a descriptor-based representation of chemical space.

Employing a methodology comparable to Reese et al.,^50^ the physicochemical space of EMA-authorized orally administered drugs was compared against four rulesets that describe oral lead-likeness and drug-likeness. These include, in order of when they were first proposed, the Ro5,^9^ Veber’s Rules,^51^ the extended Rule of Five (eRo5),^52^ and the contemporary Rule of Five (cRo5).^50^ The parent form of the drug was used in the calculation of all descriptors, with the exception of molecular weight where both the parent drug and the original drug substance less any solvate moieties was used.

### Excipient Selection

Exploratory hypothesis testing was performed to examine the effect of various descriptors of active ingredients on excipient selection. The six descriptors used in the drug likeness rulesets were calculated for all free drugs (i.e., excluding active substances formulated as salts and solvates) formulated as a tablet which is not specified to have a modified release profile (*n* = 160). For each excipient formulated with at least ten active ingredients (*n* = 33), a two-sided Mann–Whitney *U* test was conducted using SciPy^53^ to compare the distribution of each chemical descriptor between free drugs formulated with the excipient and those not formulated with it. This test was chosen due to the small sample size associated with some excipients, the presence of outliers, and the potential for deviations from normality and homoscedasticity on some splits of the data. The Benjamini–Hochberg procedure was applied to control for the false discovery rate, with both unadjusted and adjusted *p*-values for all tests reported on the GitHub repository. The significance level was set to 0.05. This procedure was chosen as it is more powerful than conservative methods to account for multiple comparisons, such as the Bonferroni correction, making it better suited for exploratory analysis.^54^

Association rule learning, an unsupervised ML method used to uncover interesting patterns in large itemset collections, was applied to the excipient data in the pharmacoinformatics database. Readers may consult the review by Naulaerts et al.^55^ for an introduction to frequent itemset mining. Briefly, the aim of association rule learning is to generate if-then rules, e.g. if copovidone is used, then polyethylene glycol is used. In this example, copovidone is termed the antecedent and polyethylene glycol is termed the consequent. The support of a rule refers to the proportion of all transactions in which an itemset occurs (eq 1).
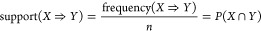
1where *X* is the antecedent, *Y* is the consequent, and *n* is the number of transactions. Confidence is the conditional probability that the consequent is true given that the antecedent is true (eq 2).
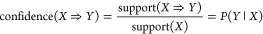
2

Lift is the ratio of the probability of the occurrence of the antecedent and the consequent together to the expected probability if they were independent (eq 3).
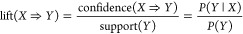
3

The apriori algorithm^56^ was implemented in Python using the arulespy library.^57^ Data from all oral immediate release tablet products, with each list of excipients being considered a transaction, was used to generate frequent itemsets, association rules, and association network graphs.

### Prediction of Oral Fraction Absorbed

Binary classification models of oral *F*_a_, using an *F*_a_ of greater than or equal to 0.8 as a cutoff, were constructed using scikit-learn^58^ in a manner broadly similar to Rath et al.^59^ The reader is directed to the GitHub repository for a complete description of the modeling process. Briefly, orally administered drugs with an associated *F*_a_ value were selected from the database and again verified to be correct. In cases where multiple *F*_a_ values were available, the greater value was taken. Drugs whose parent form contained the nonbonded character, denoted in the SMILES string as a dot, were excluded, resulting in 122 valid *F*_a_ values. Data was partitioned into training and testing sets with a test size of 0.2. The train-test split was random but stratified by *F*_a_ class due to the class imbalance, as only 26 values had an *F*_a_ below 0.8. A range of feature sets as summarized in Table 1 were considered, encompassing fingerprints, two-dimensional, and three-dimensional descriptors. Energy minimization was performed using the RDKit implementation of the MMFF94 force field^60^ before calculation of the three-dimensional descriptors. The modelability index,^61^ a simple measure of class structure which describes the expectation of obtaining a binary classifier with a balanced accuracy above 0.7 for a given descriptor set, was calculated using data from both the training and testing sets, but was not used for feature set selection. For Lipinski, RDKit2D, and Mordred descriptor sets, the data was first scaled to zero mean and unit variance using values from all 122 drugs for calculation of the modelability index only. Modelability indices above 0.65 indicate that the data is modelable.

**Table 1:** Descriptor Sets Used to Model Oral *F*_a_^a^

name	description	MI
MACCS	166 molecular access system keys	0.647
ECFP6^62^	2048-bit extended connectivity fingerprints (radius = 3)	0.484
Lipinski	18 RDKit.Chem.Lipinski descriptors	0.668
RDKit2D	200 RDKit 2D descriptors implemented by Descriptastorus	0.663
Mordred^63^	>1800 2D and 3D descriptors	0.528

a MI = modelability index.

A grid search of hyperparameters was performed for decision trees, random forests, adaptive boosting, and gradient boosting machines. Rescaling of the data in this case was not required as the algorithms considered are invariant to scaling. Balanced accuracy, which is the arithmetic mean of sensitivity (the true positive rate) and specificity (the true negative rate), was selected to measure performance due to the skewed class proportions. 10-fold cross-validation was used to optimize the hyperparameters of each model by grid search. In the case of decision trees, for example, the values of five different hyperparameters (splitting criterion, maximum depth, maximum number of features, minimum samples required to split, and minimum samples allowed in a leaf node) were selected by grid search. Folds were stratified by *F*_a_ class to ensure each fold was representative of overall class proportions. The search strategy in each case is provided in the code. The best hyperparameter configuration was selected on the basis of average balanced accuracy across the ten folds, with the standard deviation of balanced accuracy values giving an indication of stability. All data in the training set was then used to refit the best performing model identified by cross-validation, and performance was assessed on the test set. Overfitting was assessed by comparing the final training and test set balanced accuracy scores.

## Results and Discussion

**Figure 4:**
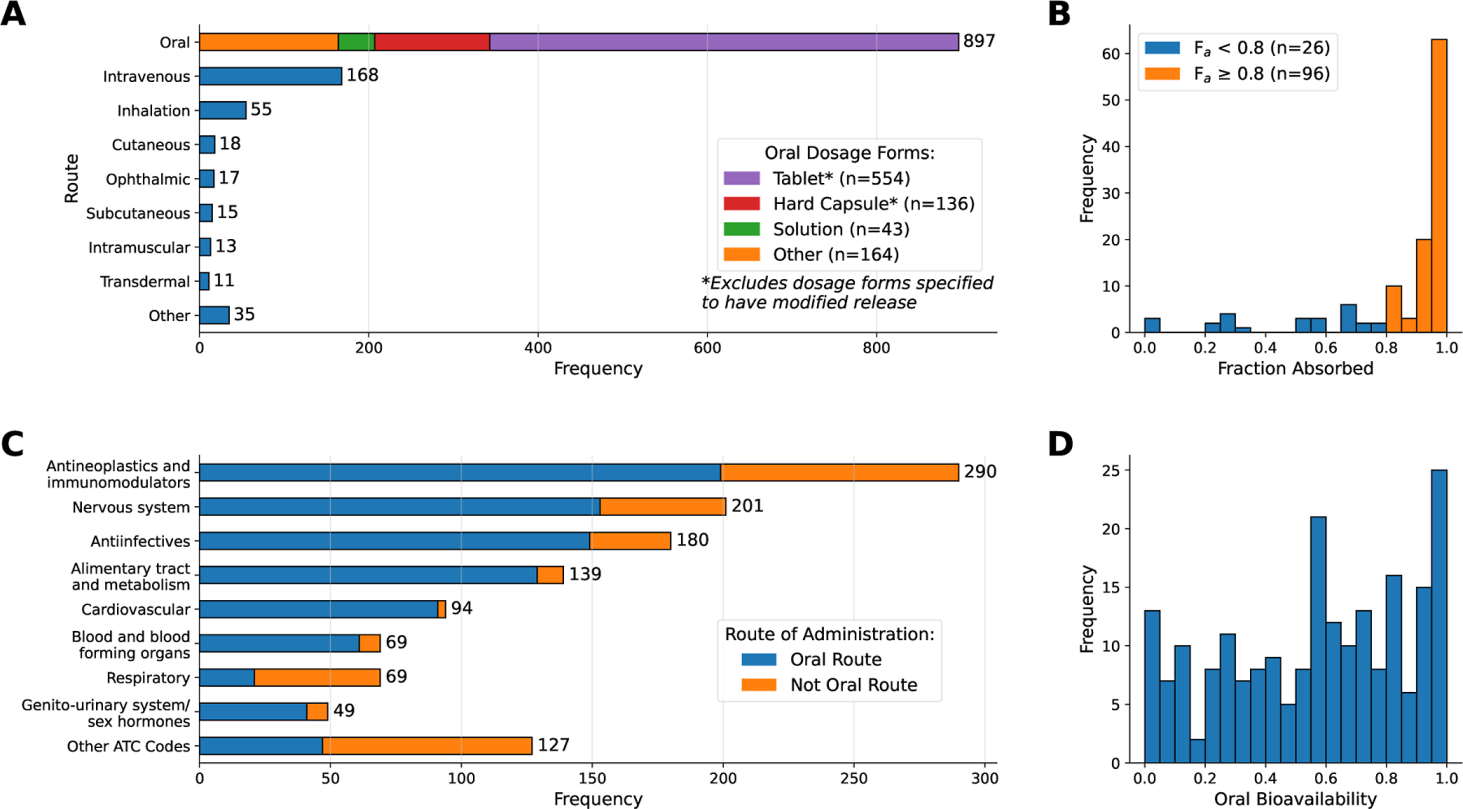
Exploratory data analysis of the pharmacoinformatics database. (A) Distribution of routes of administration for 1229 formulations. The frequency of dosage forms in the case of orally administered products is also shown. (B) Histogram of *F*_a_ values for 122 orally administered drugs. The data is partitioned into two classes at a cutoff of 0.8. (C) ATC Level One classifications for 1218 formulations with an assigned code, highlighting the proportion of formulations in each class that are orally administered. (D) Distribution of oral bioavailability for 214 orally administered drugs.

Despite the growth of data-driven modeling in the pharmaceutical sciences in recent years, the availability of large drug product libraries to support drug product design remains limited. Efforts toward database curation have focused primarily on the drug substance rather than the drug product and have been constructed manually rather than programmatically. This study therefore investigated the feasibility of a reproducible database construction pipeline, where web scraping and automated processing would streamline data retrieval. The final database captures 588 drug substances and 1229 formulations. Drugs from each of the 14 anatomical and pharmacological groups defined at Level One of the Anatomical Therapeutic Chemical (ATC) classification System^64^ are included. Figure 4 displays the distributions of routes of administration, therapeutic class, formulation types, and oral pharmacokinetic parameters. An interactive Streamlit app that allows users to explore the database is available via the GitHub repository.

### Coverage of Chemical Space

**Figure 5:**
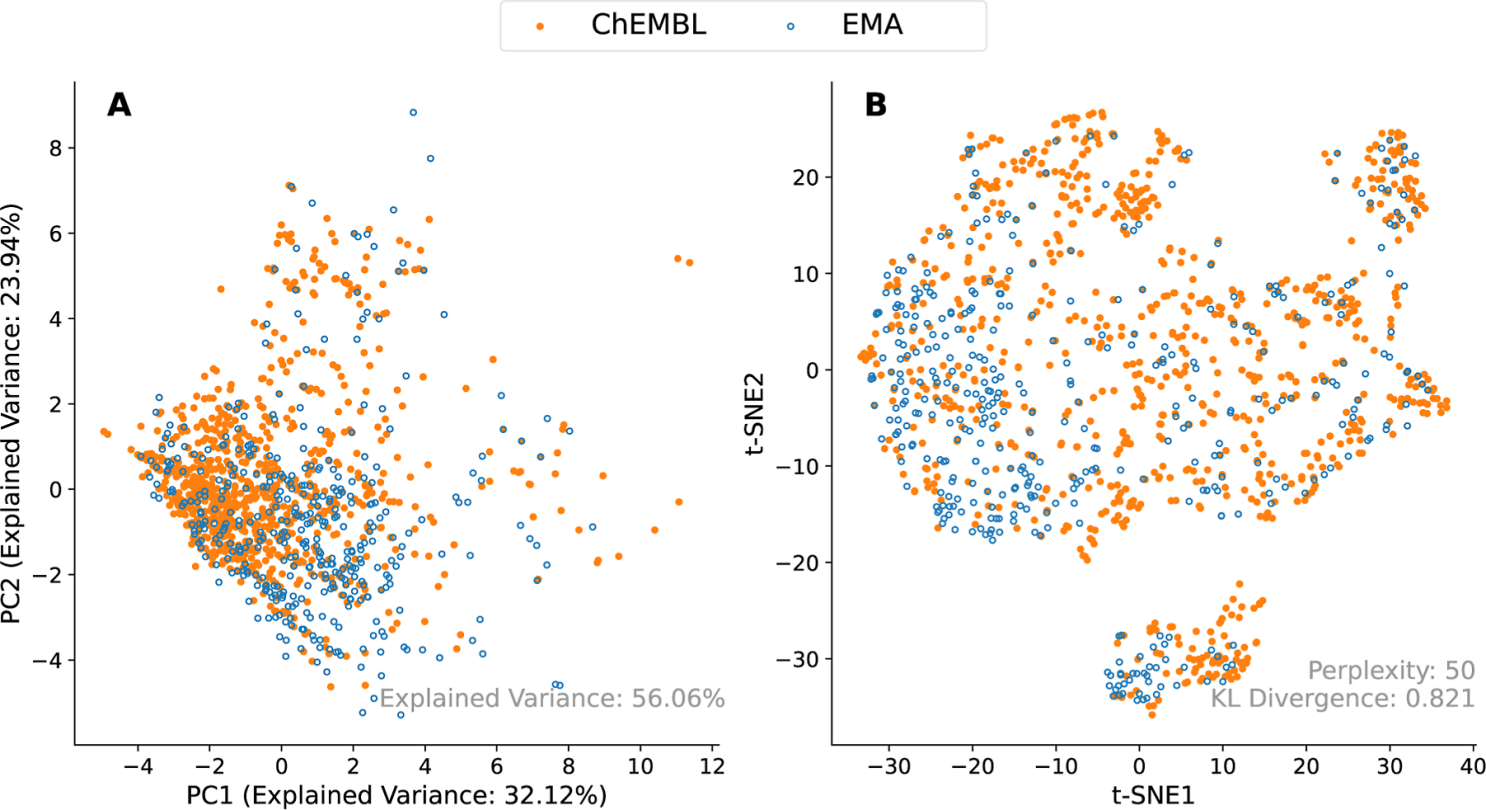
(A) PCA applied to all descriptors in the rdkit.Chem.Lipinski module. (B) *t*-SNE plot of pairwise Tanimoto distances, as calculated using MACCS keys. The overlap in chemical space covered by drugs in the pharmacoinformatics database when compared to all drugs that have reached Phase IV as captured by ChEMBL is evident in both plots.

Dimensionality reduction techniques confirm that the chemical space covered by orally administered active substances in the pharmacoinformatics database overlaps with the chemical space of orally administered active substances that have reached Phase IV of development in the ChEMBL database (Figure 5). The PCA plot showing the first two principal components shows no clear separation between the two groups of compounds. The overlap is also evident in the structure-based *t*-SNE projection which again demonstrates similarity of chemical space. The Kullback–Leibler (KL) divergence,^65^ which assesses how accurately the *t*-SNE representation preserves the structure of the original high-dimensional data, measured 0.821. Although a KL divergence near zero may be desired for clustering tasks, a value of less than one still indicates that the embedding preserved some of the structure of the original data. In addition, the distribution of the data is in line with previous similar use cases of *t*-SNE for demonstrating chemical diversity, including by Rao et al.^49^ where drugs approved and discontinued by the FDA are plotted on the same *t*-SNE plot. Despite the fact that the EMA’s centralized procedure is required for the approval of drug products in some therapeutic areas and not others, these findings indicate that the pharmacoinformatics database comprehensively covers the diversity seen in the chemical space of all orally administered drugs.

### Assessment of Drug-Likeness

**Figure 6:**
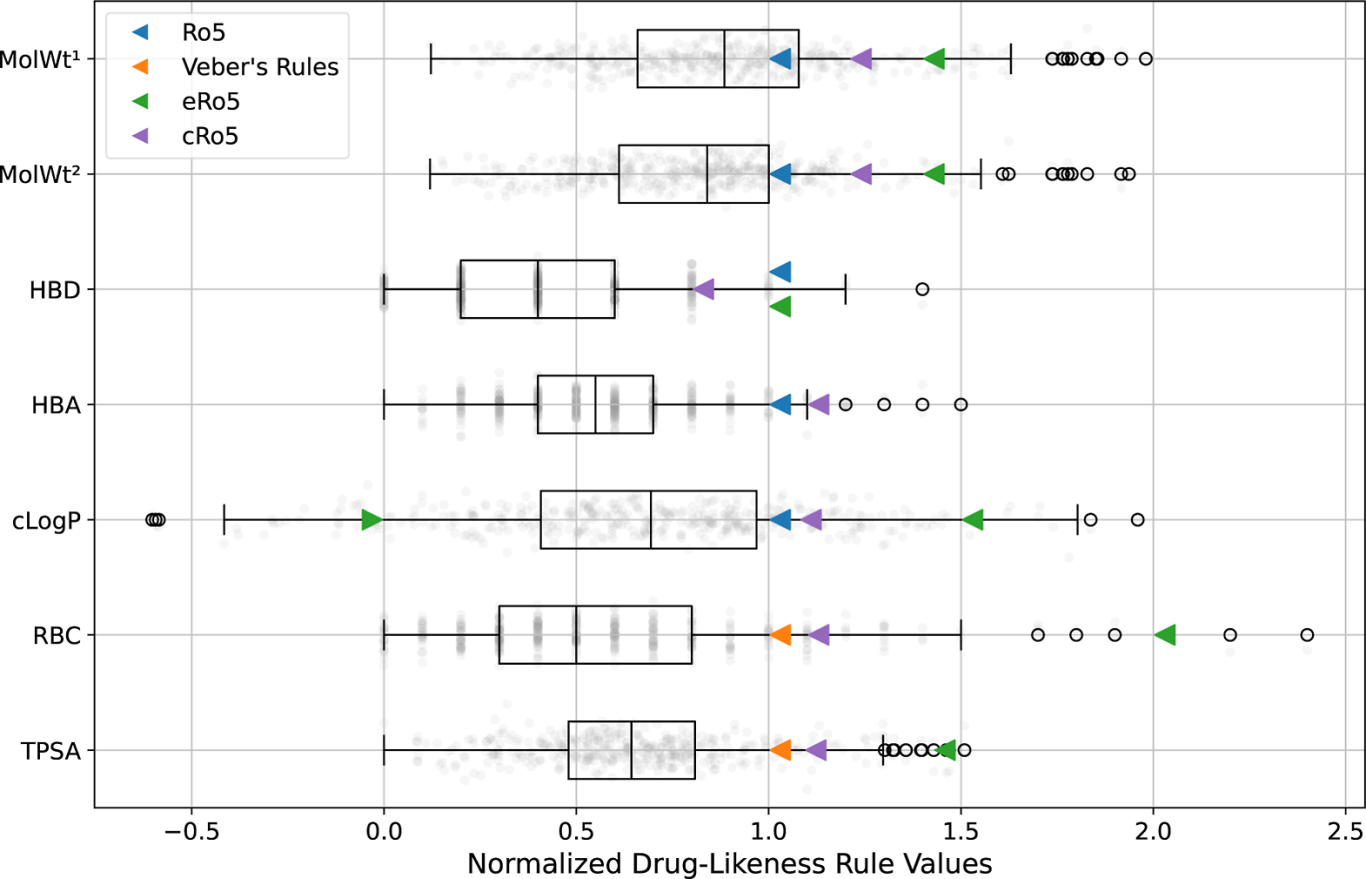
Box plots of the physicochemical properties used in drug-likeness assessments for orally administered drugs. Each property is normalized against the upper limits in the Ro5 [in the case of molecular weight (MolWt), hydrogen bond donor count (HBD), hydrogen bond acceptor count (HBA), and Wildman–Crippen log *P* (*c* log *P*)^69^ or Veber’s Rules [in the case of rotatable bond count (RBC) and topological polar surface area (TPSA)]. The triangles represent the upper and lower bounds associated with each ruleset. For example, the blue triangle on the *c* log *P* line represents the Ro5 upper *c* log *P* limit of 5. MolWt^1^ refers to the molecular weight of the active substance with any solvate moieties removed, divided by the number of parent drug molecules in the SMILES string (see Figure 2.) MolWt^2^ refers to the molecular weight of the parent drug. Note that in cases where more than one valid multicomponent active substance was available for a single parent drug to calculate MolWt^1^, the SMILES associated with the active substance’s first occurrence in the database was selected.

Figure 6 illustrates the physicochemical properties of orally administered drugs on a scale normalized against either the Ro5 or Veber’s Rules. While every ruleset captures the interquartile range of each descriptor included when the parent drug is considered, no ruleset successfully captures the total chemical space of orally administered compounds. Of 394 orally administered parent compounds, only 64.0% conform perfectly to the Ro5, 82.0% to Veber’s Rules, 84.8% to the eRo5, and 72.1% to the cRo5, where all criteria in each ruleset are considered. 16.2% of parent drugs violate more than one criterion of the Ro5. When the molecular weight of the administered active pharmaceutical ingredient less any solvent moieties is considered, as done in some sections of the original Ro5 analysis, the interquartile range is no longer captured by a limit of 500 g mol^–1^, with 137 compounds exceeding this recommendation compared to 99 parent drugs. As our database was limited to drugs whose parent form has a molecular weight of below 1000 g mol^–1^, classic examples of beyond Ro5 drugs^52^ such as cyclosporin are not included and, as such, this study likely overestimates the proportion of drugs conforming to drug-likeness rules. This analysis therefore corroborates previous work which indicates that the orally druggable chemical space cannot be reduced to a simple mnemonic and strict adherence to such rulesets during drug design and lead selection is not advised.

In line with previous findings,^50,66^ this study demonstrates the importance of a nuanced approach to drug design which is not led prescriptively by rulesets of oral druggability. Kenny and Montanari^67^ and Shultz^68^ emphasize the shortcomings of using property-based rulesets to define a chemical space for oral drugs, and question the existence of drug-like properties. The results presented in Figure 6 show that drugs only tend to fall into the filters defined by Lipinski and Veber, but that this is not always the case. A set of nondrug small organic molecules is needed to determine whether these properties truly capture drug-likeness in order to overcome selection bias or the base rate fallacy,^68^ which is beyond the scope of this paper. The novelty of this study lies instead with the streamlined curation and analysis process, rather than the analysis itself which has been previously reported. While the extraction of a valid active substance name from the raw regular expression match was time-consuming, the integration of chemical structure via the SMS and PubChem and subsequent automated curation was seamless and greatly reduced the likelihood of human error. Further, by verifying that each parent drug structure matched to a ChEMBL entry programmatically, and flagging those which did not for manual curation, the potential for an error in either PubChem or the SMS to be integrated into the database is mitigated, albeit not eliminated.

### Excipient Selection

Previous data-driven approaches have primarily focused on the selection of formulation strategy rather than the selection of excipients.^70−72^ In this study, formulation design was explored at the individual excipient level using two data analysis techniques. Exploratory hypothesis testing was employed to identify links between drug properties and excipient selection. Association rule learning was applied to discover patterns in excipient selection independent of the active ingredient.

**Figure 7:**
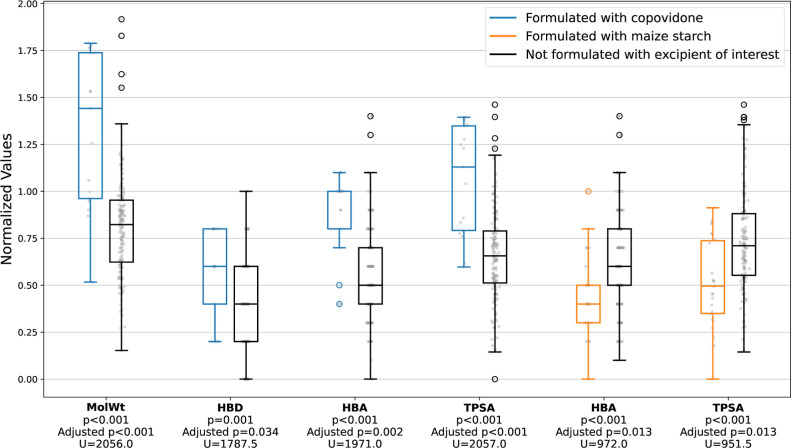
Box plots comparing the distribution of various descriptors associated with drug-likeness, with the data split on whether the drug was formulated with the excipient of interest. The scale has been normalized against the upper limits of the Ro5 in the case of MolWt, HBD, and HBA, and against the upper limit of Veber’s Rules in the case of TPSA.

Of 198 hypothesis tests performed, 27 had a *p*-value of less than 0.05. Only six adjusted *p*-values, however, had values which indicated significance. Four of these values were associated with copovidone, while two were associated with maize starch. It is important to note that the purpose of exploratory hypothesis testing is to search for patterns, and therefore the lack of a detected association does not imply that no trend exists. The distributions of MolWt, HBA, HBD, and TPSA for drugs formulated with copovidone were different from that of drugs not formulated with the excipient, with Figure 7 indicating that values were generally larger in the copovidone group in all cases. This is not surprising given that copovidone is used extensively as the polymeric carrier in amorphous solid dispersions.^70^ Of the 17 drugs formulated with copovidone, ten are antivirals used in the treatment of hepatitis C or HIV, a class of drugs well-documented to present absorption challenges.^70,73^ Conversely, the distributions of HBA and TPSA for the 26 drugs formulated with maize starch were generally lower than the corresponding distributions for drugs not formulated with this excipient. Maize starch is low-cost with an extensive history of use in conventional formulations, thereby making it an appealing excipient for drugs that do not require bioenabling technologies for commercialization.^74^ In both cases, the variety in marketing authorization holders responsible for developing the formulations indicate that the trends seen are unlikely to be a result of company preference for a given excipient.

**Figure 8:**
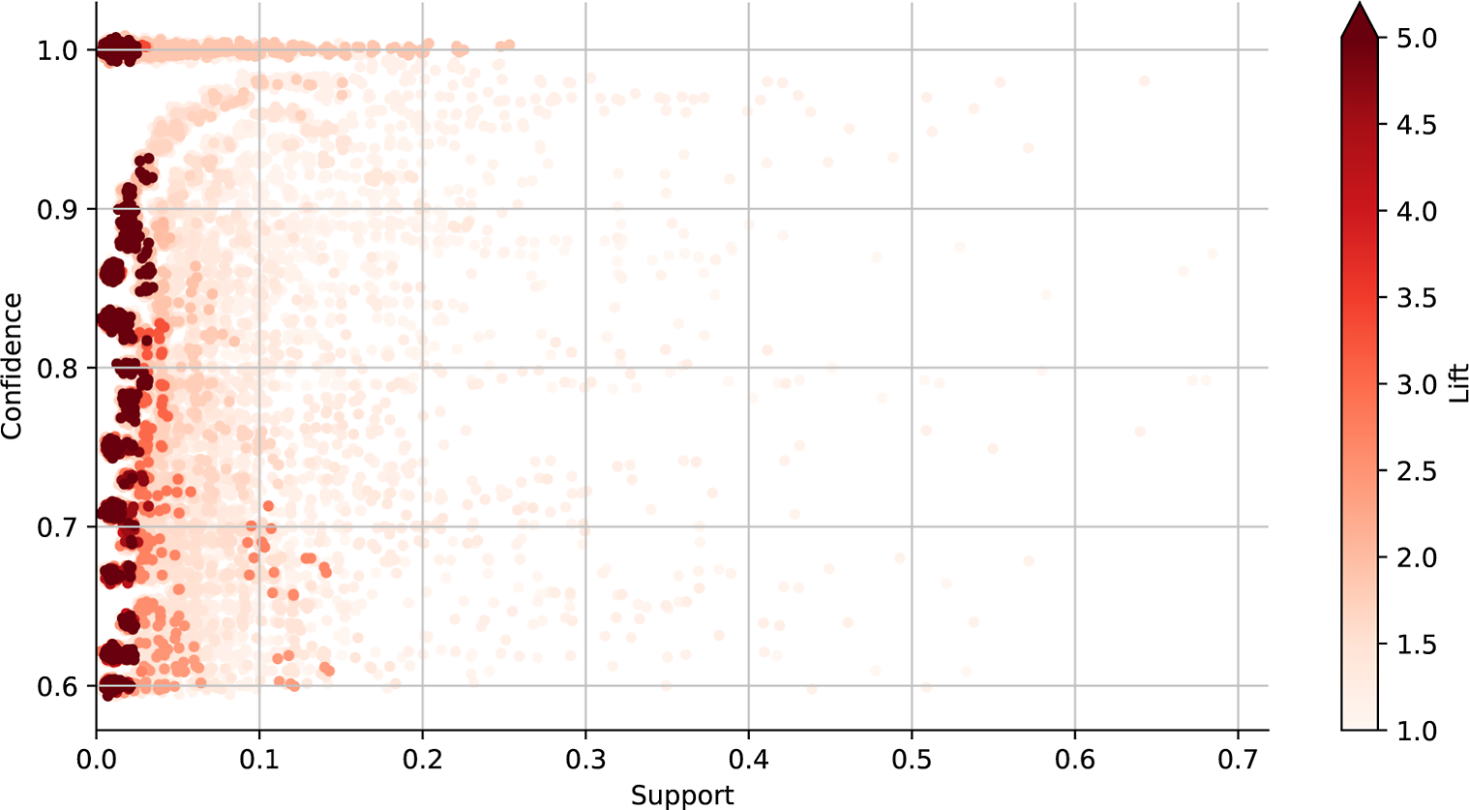
Scatter plot displaying the trends in support, confidence, and lift for 55,650 rules identified by association rule mining. Support indicates the frequency of the itemset, while confidence and lift are further filters of rule interestingness. Support and confidence thresholds of 0.01 and 0.6 respectively were applied. Only rules with a lift above 1, indicating a positive association between the antecedent and the consequent, are displayed.

Association rule learning discovered 55,650 rules that meet minimum thresholds for support, confidence, and lift, as indicated in Figure 8. The reader is directed to the GitHub repository to access an interactive table to explore association rules. The high number of identified rules is not unexpected given the combinatorial explosion in the number of possible itemsets and association rules as the number of excipients in the controlled vocabulary increases. Webb^75^ indicates that the risk of identifying spurious association rules in real-world data is “extreme” due to the large search space of pattern discovery algorithms, notwithstanding the potential for redundancy.^76^ The number of generated rules can be constrained by increasing the support and confidence thresholds. Increasing support beyond the 0.01 threshold in this case is not appropriate given that many excipients of biopharmaceutical interest have low support. Figure 8 shows many low-support high-confidence rules, which also tend to have high lift. This reflects the fact that for real-world transactional data lift and support are generally inversely related (i.e., excipients used less frequently are more likely to have high-lift rules associated with them).^57^

**Figure 9:**
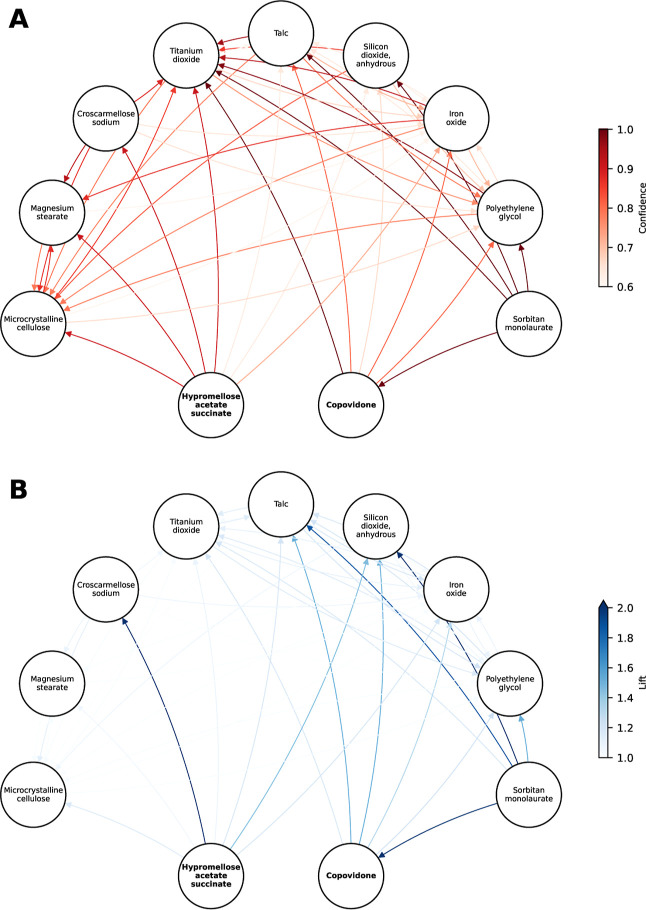
Network graphs of selected association rules. Displayed rules involve either hypromellose acetate succinate or copovidone, have one antecedent and one consequent, and have a confidence greater than 0.6 and a lift greater than one. Rules between excipients in the network are included. (A) Shows edges colored by confidence and (B) shows edges colored by lift. Arrows point toward the consequent.

A complex network of 55,650 association rules is not interpretable. As such, Figure 9 shows an association network graph for two excipients of biopharmaceutical interest due to their use as polymers in amorphous solid dispersions:^77^ hypromellose acetate succinate and copovidone. The analysis of a subset of rules in association rule mining is standard to facilitate targeted evaluation.^78^ Only rules with one antecedent and one consequent are shown for simplicity. Although confidence estimates the conditional probability of the consequent given the antecedent (eq 2), lift compares this conditional probability to the expected probability if the antecedent and the consequent were independent (eq 3). Only a small number of the rules with high confidence in Figure 9A have a co-occurrence beyond what might be expected by chance, which is also reflected in Figure 9B. Two high lift rules, namely hypromellose acetate succinate ⇒ croscarmellose sodium (lift = 2.06) and sorbitan monolaurate (Span20) ⇒ copovidone (lift = 19.62), were identified. These association rules have a naturally meaningful interpretation. In the case of the former, the use of the superdisintegrant croscarmellose sodium in solid dispersion formulations is well documented.^79−81^ This pattern was obvserved in ten formulations containing 12 drug substances and by six marketing authorization holders. Although the copovidone ⇒ sorbitan monolaurate combination was only seen in five tablets, all of which contained ritonavir either alone or with another drug, the rule has a similar meaningful mechanistic interpretation.^82,83^Figure 9 illustrates that even association rules with high lift and confidence above the support threshold are not necessarily meaningful, and that domain expertise is needed to interpret the patterns identified.

Reducing the granularity of excipient data through merging and simplification is crucial to enable data analysis. Excessive detail in the description of excipients would both greatly increase the number of possible rules and necessitate a reduction in minimum support thresholds which are already set to 0.01. Lowering this threshold further can lead to the identification of a greater number of spurious patterns and therefore reduce the quality of analysis. This approach highlights broader patterns in excipient selection and is not necessarily the most suitable technique for guiding the formulation of a specific challenging drug. While the analysis does not consider details such as the proportion or grade of excipients, frequent itemset mining can still support rational formulation design by highlighting promising excipient combinations. Association rule-based recommendation systems for excipient selection, similar to those which have been demonstrated to be successful for pharmaceutical portfolio management,^84^ could encourage a more algorithmic approach to formulation design. In this regard, the interactive Streamlit app serves as a basic recommender system.

### Prediction of Oral Fraction Absorbed

Binary classifiers of ADMET end points have the potential to prioritize drug candidates with favorable properties for synthesis and development.^59,85^ Given the importance of *F*_a_ from a biopharmaceutics perspective and the growing absorption issues associated with drugs in the development pipeline, *F*_a_ is an attractive end point to include during molecule prioritization to further reduce the risk of compound attrition. Binary classifiers were therefore constructed to predict oral *F*_a_ using only inputs calculated from the chemical structure of the parent form of the drug included in the dosage form. *F*_a_ was partitioned into two classes as indicated in Figure 4B. The cutoff of 0.8 was chosen to maintain consistency with models reported by Rath et al.^59^ and Newby et al.^86^ The aim of this analysis was 2-fold: to determine the modelability of *F*_a_ directly from chemical structure, and to assess whether data from regulatory submissions is sufficiently accurate and complete to support predictive modeling.

Table 2 shows the best performing classifier for each descriptor set, as determined by the average balanced accuracy across ten folds of cross-validation. The decision tree classifier which used RDKit2D descriptors as input achieved a balanced accuracy of 0.725, a sensitivity of 0.85, a specificity of 0.6, a positive predictive value of 0.89, and a negative predictive value of 0.5, thus satisfying the acceptability criteria proposed by Rath et al.^59^ (listed in Table 2.) The modelability index^61^ of this data set measured using RDKit2D descriptors was 0.663, which is above the 0.65 threshold for modelability. The difference between the balanced accuracy on the training set (0.902) and test set (0.725) indicates some degree of overfitting. The standard deviation in cross-validation scores of 0.188 suggests sensitivity to variations in data splitting. Despite this, the consistency between test set and mean cross-validation balanced accuracy (0.743) implies a reasonable generalization capacity.

**Table 2:** Summary of Performance of Five Descriptor Sets in *F*_a_ Classification Ranked by Cross-Validation Balanced Accuracy^a^

descriptor	classifier	cross-validation BA (SD)	train BA	test BA	acceptable^b^
RDKit2D	decision tree classifier	0.743 (0.188)	0.902	0.725	yes
Lipinski	gradient boosting machine	0.696 (0.153)	1.000	0.675	no
Mordred	decision tree classifier	0.695 (0.104)	0.855	0.575	no
MACCS	random forest classifier	0.683 (0.105)	0.810	0.650	no
ECFP6	decision tree classifier	0.642 (0.148)	0.855	0.375	no

a BA = balanced accuracy, SD = standard deviation.

b An acceptable model is one which has a balanced accuracy of ≥0.6 and all of positive predictive value, negative predictive value, sensitivity, and specificity ≥0.5 on the test set.^59^

The decision tree was constrained to a maximum depth of 8, was limited to considering a maximum of the square root of the number of features at each split, and required 8 samples in order to split an internal node. The optimal cross-validation balanced accuracy was observed when the minimum number of samples per leaf was set to one. Shannon entropy was used to evaluate splits. The tree which was fit on all training data is reasonably simple with ten leaves. The root node was split on the zero-order valence number connectivity index, ^0^χ^*n*^, indicating that this descriptor provided the greatest reduction in Shannon entropy. Topological indices^87^ have been applied widely to physicochemical and biological property prediction tasks, with ^0^χ^*n*^ and other zero-order connectivity indices incorporated in successful models of intrinsic solubility^88^ and crystallization propensity.^89^ In the *F*_a_ data set, ^0^χ^*n*^ has an almost perfect monotonic relationship with other RDKit2D descriptors, including molecular weight and other zero-order connectivity indices. Therefore its selection was not strictly deterministic but rather due in part to the randomness associated with feature selection.

Previous cheminformatics-driven binary classifiers of *F*_a_, including by Hou et al.^90^ and Pires et al.,^91^ have been reported with higher balanced accuracy scores of up to 0.984 on the test set. This paper, however, differs by including drugs which excluded by these studies (e.g., drugs absorbed by carrier-mediated transport and those showing dose-limited or dependent absorption), and by considering only the highest strength clinical formulation. These studies also had more data available as it was collected from many sources, including a highly curated 241 compound data set reported by Zhao et al.^92^ Deconinck et al.^93^ developed decision trees using a training set of 141 drugs and drug-like compounds, and then tested the performance on 27 unseen compounds. All molecules in the test were well-absorbed. The number of misclassified compounds in the test set ranged between three and six depending on the model, which is comparable to the performance observed in this study where five out of 25 molecules were misclassified in a test set which also included poorly absorbed drugs. Newby et al.^86^ integrated experimental data, including permeability measured on cell lines, solubility, dose number, and melting point, with chemical descriptors for binary classification of *F*_a_ by decision trees using a cutoff of 0.8. Expectedly, models with experimental data yielded better performance, though in many cases the difference was small. A tree built using literature cell line permeability and molecular descriptors for 356 training compounds, for example, achieved a test set sensitivity of 0.593 and specificity of 0.875 on 77 compounds. The performance achieved by our model is further comparable to imbalanced ADMET classification tasks which used EPAR and/or FDA label data.^94,95^

Klingspohn et al.^96^ define two scenarios in which molecules may be associated with a high probability of misclassification: lack of similarity to molecules in the training set and proximity to the decision boundary. There is large diversity in the chemistry of the drugs in the training set, which makes the definition of an applicability domain difficult. Regarding the decision boundary, 32 drugs fall within 0.1 of the decision boundary of 0.8, with three drugs having an *F*_a_ of 0.8 exactly. In addition, the label noise of *F*_a_ is significant. Not only is it a property which depends on dose, solid state, and delivery system, there is the additional noise associated with large intra- and interindividual variability. All *F*_a_ values in the database are reported as point values rather than ranges, and it is possible that the range of possible *F*_a_ values may overlap with the decision boundary. Anagrelide hydrochloride was assigned to the minority class on curation as it has an absorption of “at least 70%···from the gastrointestinal tract,” despite the language indicating that *F*_a_ may be above 80% in some individuals. This problem is not exclusive to *F*_a_ prediction and the challenges associated with discretizing continuous variables for ADMET prediction are well documented.^97,98^

It remains unclear to what extent the skewness in *F*_a_ values (Figure 4B) is a true representation of the underlying data. Complete oral absorption can be inferred from 100% absolute oral bioavailability (Figure 4D), but not any other bioavailability value as it is not possible to differentiate between the fraction not absorbed and the fraction metabolized. These heavily skewed class proportions exacerbate the challenges associated with classification, especially in accurate assignment to the minority class. Hou et al.^90^ similarly observed skewness toward well-absorbed compounds yet had data on a sufficient number of poorly absorbed compounds to place the cutoff at 0.3. Furthermore, different salt and solvate forms of the same drug often had equivalent *F*_a_ values quoted in the EPARs, making it impossible to attribute a gain or loss in *F*_a_ to the presence of a hydrate or counterion. Four different salt forms of clopidogrel are all associated with an *F*_a_ of 0.5. An equivalent phenomenon is observed for excipients, where generic products are formulated in many cases to be bioequivalent to the reference product and therefore the same *F*_a_ is quoted.

Additionally, not all regulatory documents discriminated between *F*_a_ and oral bioavailability. Opicapone is quoted in its SmPC to have an “absorption” of approximately 20%, but it is later stated that a combined 28.7% of the radioactivity of a single ^14^C-opicapone dose was excreted in urine and expired air. Clinical pharmacokinetic studies of opicapone demonstrate that the fraction absorbed is much higher, likely above the decision boundary of 0.8 selected for modeling.^99,100^ Given that this study did not intend to train a classifier immediately suitable for use in production but rather aimed to demonstrate the potential of the underlying data to support modeling efforts, the classifier was not retrained with the correct value for opicapone. The database has, however, been annotated to highlight the ambiguous wording. In contrast, the statement in the SmPC for raloxifene hydrochloride that “60% of an oral dose is absorbed” refers to *F*_a_ as the absolute bioavailability is later specified to be 2%. Data on the oral formulation-dependent pharmacokinetic parameters was often omitted. In many instances the absolute bioavailability of an orally administered drug product could not be determined as no equivalent intravenous formulation was available. The pharmacokinetic parameters were also frequently only reported with respect to the diseased population which can result in significant changes to drug disposition, as is the case with atazanavir and lenacapavir.

Despite the challenges associated with absorption as an ADMET end point, this work demonstrates the potential of a binary *F*_a_ classifier to support compound prioritization tasks, even in the absence of knowledge regarding dose-limited absorption and carrier-mediated transport. The performances indicate limitations in the models, including a propensity for the classifiers to overfit on the training set and instability on cross-validation. Nonetheless, the aim of this study was to assess the feasibility of an *F*_a_ classifier that can be used in combination with many end points, capturing potency, synthetic accessibility, and ADMET properties, for multiobjective optimization tasks at early stage drug development. While further data and validation are necessary to confidently integrate *F*_a_ into prioritization frameworks, these findings provide promising indications of the modelability of *F*_a_, independent of dose, solid form, and delivery system.

### Future Outlook

This study demonstrated the benefits of a drug product database which integrates various sources of existing curated data. While extensive cleaning of semistructured regulatory documents was required, a growing infrastructure for the programmatic retrieval of data from regulatory authorities may eliminate this need entirely. Such a mechanism already exists for FDA data in the form of openFDA.^101^ At the time of writing, the EMA is developing an application programming interface for the ePI and has indicated that guest access will be available. Programmatic retrieval, however, does not eliminate the other challenges associated with regulatory data. Even with the use of an application programming interface, pharmacokinetic information is often embedded in text^16^ and it was demonstrated in this paper that the parameters are not always accurate or available. As discussed, the description of excipients provided in regulatory documents was often too granular to support modeling efforts. Therefore, curation of retrieved data was still necessary. Conversely, Hancock and Goldfarb^102^ contend that the existing descriptions are not granular enough to describe the physical characteristics and performance of excipients. The same authors propose the development of a systematic excipient taxonomy to permit the unambiguous identification of excipients. In the context of data-driven formulation development, a hierarchical excipient taxonomy would be particularly advantageous by allowing for the control of granularity through the automated grouping of excipient classes. An internationally recognized excipient taxonomy would enhance the semantic interoperability of drug product databases among regulatory authorities, offering a more integrated approach than the current SMS central dictionary.

The adaptation of the work in this paper to large molecule formulations warrants additional exploration, as does the potential for real-world drug product data to serve as training material for generative artificial intelligence. The analysis of formulation data through exploratory hypothesis testing and association rule learning may be applied to larger databases of authorized drug products, such as the FDA Pillbox database,^103^ and experimental formulations, such as the self-emulsifying formulations described by Zaslavsky and Allen.^22^ Further interrogation of *F*_a_ as a modelable property for compound prioritization tasks at early stage development is needed and should be supported by the increased availability of machine-readable data.

Efforts toward interoperability have been driven by both design on the regulatory side, and extensive post hoc mapping.^27^ A global data model for drug product information which is linked to external curated databases at the design stage would largely eliminate the need for manual integration. Such a data model would support multiple needs which are not limited to predictive modeling, such as clinical practice pharmacovigilance, and regulatory submissions.^31^ Implementing the Industry/Pharma 4.0 strategies of data maturity and data integrity in the context of information management promises to expedite pharmaceutical development by making predictive modeling more efficient.

## Conclusion

As the volume of data available to pharmaceutical scientists grows exponentially year-on-year, there is clear potential for real-world data to inform future drug product development. A comprehensive and machine-readable library of existing drug products which links many aspects of drug product design, including the active substance(s), excipients, pharmacokinetic performance, therapeutic indication, and authorization status, was lacking. This research describes the successful use of web scraping and programmatic data retrieval to expedite the database curation process with a reproducible workflow. The application of this database to fundamental tasks such as drug likeness evaluation, excipient selection, and prediction of oral *F*_a_ was demonstrated. While these results support the case for data-informed drug product development, data-driven models are limited by the shortcomings of the underlying data. Missing values and logical inconsistencies of the values reported in the regulatory datasphere impede modeling efforts. This study highlights the potential benefit of a comprehensive drug product database. A global data model of drug information which prioritizes public accessibility, machine-readability, and accuracy is needed from the regulatory side in order for such a pharmacoinformatics database to be truly self-propagating.
